# Method validation and preliminary qualification of pharmacodynamic biomarkers employed to evaluate the clinical efficacy of an antisense compound (AEG35156) targeted to the X-linked inhibitor of apoptosis protein XIAP

**DOI:** 10.1038/sj.bjc.6603220

**Published:** 2006-06-27

**Authors:** J Cummings, M Ranson, E LaCasse, J R Ganganagari, M St-Jean, G Jayson, J Durkin, C Dive

**Affiliations:** 1 Clinical and Experimental Pharmacology, Paterson Institute for Cancer Research, University of Manchester, Wilmslow Road, Manchester M20 4BX, England, UK; 2 Department of Medical Oncology, Christie Hospital NHS Trust, Wilmslow Road, Manchester M20 4BX, England, UK; 3 Aegera Oncology Inc., CHEO Research Institute, 401 Smyth Rd, Ottawa, Ontario, Canada K1H 8L1

**Keywords:** M65 Elisa, M30 Apoptosense, quantitative RT–PCR, validation, stability, biomarker variability

## Abstract

Data are presented on pharmacodynamic (PD) method validation and preliminary clinical qualification of three PD biomarker assays. M65 Elisa, which quantitates different forms of circulating cytokeratin 18 (CK18) as putative surrogate markers of both apoptotic and nonapoptotic tumour cell death, was shown to be highly reproducible: calibration curve linearity *r*^2^=0.996, mean accuracy >91% and mean precision <3%, *n*=27. Employing recombinant (*r*) CK18 and caspase cleaved CK18 (CK18 Asp^396^ neo-epitope) as external standards, kit to kit reproducibly was <6% (*n*=19). rCK18 was stable in plasma for 4 months at −20°C and −80°C, for 4 weeks at 4°C and had a half-life of 2.3 days at 37°C. Cytokeratin 18 Asp^396^ NE, the M30 Apoptosense Elisa assay antigen, was stable in plasma for 6 months at −20°C and −80°C, for 3 months at 4°C, while its half-life at 37°C was 3.8 days. Within-day variations in endogenous plasma concentrations of the M30 and M65 antigens were assessed in two predose blood samples collected from a cohort of 15 ovarian cancer patients receiving carboplatin chemotherapy and were shown to be no greater than the variability associated with methods themselves. Between-day fluctuations in circulating levels of the M30 and M65 antigens and in XIAP mRNA levels measured in peripheral blood mononuclear cells by quantitative (q) RT–PCR were evaluated in two predose blood samples collected with a 5- to 7-day gap from 23 patients with advanced cancer enrolled in a phase I trial. The mean variation between the two pretreatment values ranged from 13 to 14 to 25%, respectively, for M65, M30 and qRT–PCR. These data suggest that the M30 and M65 Elisa's and qRT–PCR as PD biomarker assays have favourable performance characteristics for further investigation in clinical trials of anticancer agents which induce tumour apoptosis/necrosis or knockdown of the anti-apoptotic protein XIAP.

AEG35156 is a second generation, mixed backbone, 19-mer antisense oligonucleotide targeting the inhibitor of apoptosis protein XIAP and is currently undergoing Phase I clinical evaluation at two centres in the UK (Ranson *et al*, 2005). Considerable evidence has now accrued through application of a broad range of technologies to a wide spectrum of *in vitro* and *in vivo* model systems to substantiate XIAP as a valid molecular target in cancer therapeutics (Lacasse *et al*, 2005; Wright and Duckett, 2005; Schimmer *et al*, 2006). A unique feature of the ongoing AEG35156 clinical studies is that a number of pharmacodynamic (PD) biomarker assays have been incorporated into the trial design in order to seek confirmatory evidence of target knockdown and concomitant downstream effects. In addition many other anticancer agents act by inducing apoptosis or necrosis and reliable biomarkers of drug effect are required (Bast *et al*, 2005; Ludwig and Weinstein, 2005).

In a recent publication, analytical method validation of three different PD biomarker assays was reported: quantitative RT–PCR (qRT–PCR) for XIAP mRNA expression, Western blot analysis for XIAP protein expression, and a novel plasma Elisa assay (M30 Apoptosense) as a surrogate marker of tumour cell apoptosis (Cummings *et al*, 2005). During that study method validation was conducted in accordance with available internationally recognised bioanalytical guidelines established by the pharmaceutical industry, but primarily utilised in drug and safety monitoring (Shah *et al*, 2000; Miller *et al*, 2001). There is a growing acknowledgement that these guidelines are not sufficiently flexible to accommodate the many different categories of PD assays that are employed during anticancer drug development of molecularly targeted agents (Lee *et al*, 2005). It is now also evident that qualification of a biomarker is a multistage process requiring a concerted team effort often paralleling the drug development cycle (Colburn, 2003; Benowitz, 2004; Ransohoff, 2004; Ludwig and Weinstein, 2005). For PD biomarker assays at least four distinct categories were identified each requiring evaluation of a different set of performance criteria: definitive quantitative (e.g. mass spectrometry), relative quantitative (e.g. Western blot analysis), quasiquantitative (e.g. qRT–PCR) and qualitative (e.g. immunohistochemistry) (Lee *et al*, 2005).

A vital component in PD biomarker validation is the establishment of its pretreatment range and inherent biovariability, in order to assess the likelihood that the technology of choice and trial design have the statistical and technological resolving power to discriminate between normal background biologic variation and a drug-induced effect (Lee *et al*, 2005). In the present paper, analytical validation of a fourth PD assay, the M65 Elisa (Kramer *et al*, 2004), is described, along with long term stability studies on the antigens for this assay as well as for M30 Apoptosense (Biven *et al*, 2003; Kramer *et al*, 2004). In addition, an evaluation of predose biomarker variability in cancer patients is presented for the M30 and M65 cell death Elisa's and a qRT–PCR method for XIAP mRNA.

## MATERIALS AND METHODS

### Reagents

**Figure 1:**
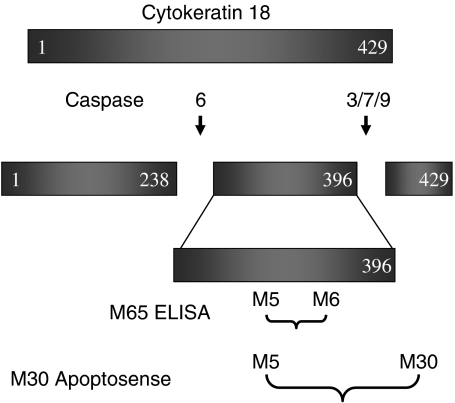
Schematic representation of the CK18 epitope map targeted by the antibodies used in the M30 Apoptosense and M65 sandwich Elisa assays. In the case of M65 Elisa the M6 antibody acts as the catcher and M5 as detection antibody. For the M30 Apoptosense assay M5 was the catcher and HRP-conjugated M30 the detection antibody.

Custom synthesised PCR primers for XIAP, the XIAP-specific Taqman probe and all other core reagents for qRT–PCR were as previously reported in full (Cummings *et al*, 2005). Staurosporine was from the Sigma Chemical Company (Poole, England, UK). Recombinant human Cytokeratin 18 (rCK18, catalogue number RDI-PRO62217) was from Research Diagnostics Inc. (Flanders, NJ, USA) and upon arrival was separated into 1 *μ*g aliquots and stored at −80°C before use. Caspase cleaved CK18 Asp^396^ neo-epitope (CK18 Asp^396^ NE, see Figure 1) was produced by incubation of MDA-MB-231/X-G4 cells (XIAP knockdown) (McManus *et al*, 2004) with 1 *μ*M staurosporine for 48 h, under conditions resulting in maximum induction of apoptosis and optimal release of the 21 kDa peptide fragment into tissue culture media (Bantel *et al*, 2004; Cummings *et al*, 2005). The conditioned media was centrifuged at 1000 **g** for 20 min to remove cellular debris and the supernatant was adjusted with fresh media to yield a stock concentration of M30 antigen of 10 000 U l^−1^, where 1 U is equivalent to 1.24 pmol of peptide (Ueno *et al*, 2003). The stock solution of M30 antigen was aliquoted and stored at −80°C before use. The M30 Apoptosense and M65 Elisa kits were both obtained from PEVIVA AB (Bromma, Sweden). All other chemicals, reagents and buffers were of the highest grade available commercially and water was purified and deionised in a Millipore Elix 3 system (Millipore, Watford, England, UK).

### Preparation of quality control samples of volunteer human plasma containing either M30 or M65 antigens for stability studies

Blood was collected from a group of healthy volunteers with informed consent in the registered phlebotomy centre of the Paterson Institute for Cancer Research, Manchester, UK. Plasma was immediately separated by centrifugation at 1000 **g** for 10 min and the supernatants pooled. To one-half of the pooled plasma was added freshly thawed rCK18 to achieve a final concentration of M65 antigen of circa 1500 U l^−1^ and to the other half of the pool was added CK18 Asp^396^ NE to achieve a final concentration of M30 antigen of circa 800 U l^−1^. Each lot of pooled plasma was thoroughly mixed and centrifuged, before aliquoting into approximately 200 individual 1.5 ml polypropylene tubes to be used as QC samples in long-term stability studies and in method validation of the M65 assay.

### Collection of predose blood samples from cancer patients for M65 and M30 Elisa analysis

Samples were utilised from two separate trials following informed consent. In the first, two predose samples were obtained from cancer patients with a 5- to 7-day gap between collections as part of a phase I trial of the XIAP inhibitor AEG35156. In this case, 5 ml of venous blood was drawn into a vacutainer tube containing heparin anticoagulant. Within 30 min of collection bloods were centrifuged at 1000 **g** for 10 min to obtain plasma and the plasma transferred to 3 ml polypropylene cyrotubes. Tubes were then frozen at −20°C within 30 min and transferred to −80°C within 3 h. In the second study, two predose samples were obtained from cancer patients within the same day. The aim of this study was to establish the baseline values and variability in endogenous plasma levels of full-length CK18 and CK18 Asp^396^ NE in patients with ovarian cancer before and during treatment with platinum based chemotherapy. The sample handling protocol was essentially the same as in the AEG35156 phase I trial. All samples from both trials were analysed within 4 months of collection.

### The M65 Elisa assay

The M65 Elisa assay is a commercially available kit based on a 96-well plate format, it consists of a seven-point calibration curve (125–2000 U l^−1^ of antigen) and certified low and high quality controls (QC, circa 200 and 1000 U l^−1^), and was operated according to the manufacturer's instructions. In brief, 25 *μ*l of sample (standard, blank, QC, or patient plasma sample) was added to each well, which was coated with a mouse monoclonal ‘catcher’ antibody that binds to an epitope on CK18 (M6 antibody, see Figure 1). Simultaneously, 75 *μ*l of HRP-conjugated monoclonal antibody (M5) solution was added to act as the detection antibody (Kramer *et al*, 2004). Samples were then incubated for 2 h at room temperature with constant shaking, after which excess unbound conjugate was removed by addition of the wash solution, times five. Colour development was achieved by incubation with 200 *μ*l of 3,3′,5,5′-tetramethyl-benzidine solution for 20 min in the dark. The reaction was stopped with 50 *μ*l of 1.0 M sulphuric acid and absorbance was finally measured in a microplate reader at 450 nm. By plotting a standard curve of known concentrations of M65 antigen standards *vs* absorbance, the amount of antigen in the QCs and unknown samples was calculated by interpolation. The M30 Apoptosense assay was performed as previously published (Cummings *et al*, 2005).

### Blood collection for RNA extraction and XIAP determination by qRT-PCR

Venous blood samples (approximately 2.5 ml) for XIAP mRNA analysis were collected from a cohort of cancer patients entered into the AEG35156 Phase I trial. Two predose samples were obtained from the patients with normally a 5- to 7-day gap between collections as part of the full PD protocol. Venous blood samples were collected into 2.5 ml PAXgene Blood RNA extraction tubes (PAXgene Blood RNA kit, PreAnalytiX/Qiagen, Catalogue Number, 762132, Valencia, CA, USA). The PAXgene tubes were inverted 8–10 times and cells lysed at room temperature for 2–24 h before being frozen at −20°C after which they were transferred to −80°C storage.

Frozen PAXgene tubes were allowed to thaw for 4 h before centrifugation at 2490 **g** for 20 min in a swing out rotor and the supernatants discarded (IEC Centra GP8R bench top centrifuge). Pellets were resuspended in RNase-free water (5 ml), further centrifuged as above and finally resuspended in 360 *μ*l buffer BR1 (PAXgene Blood RNA kit, PreAnalytiX/Qiagen) by vortexing. Tubes were then incubated for 10 min at 55°C in a 280 g shaker-incubator with 40 *μ*l Proteinase K and 300 *μ*l buffer BR2. At the end of the incubation 350 *μ*l of 100% ethanol was added and the total sample was then applied on to the PAXgene extraction column together with 350 *μ*l of buffer BR3 and centrifuged at 8000 **g** for 1 min in a minifuge discarding the flow through waste. Next the extraction columns were incubated with DNase I (10 *μ*l DNase I stock solution to 70 *μ*l Buffer RDD) for 15 min at room temperature. Extraction columns were then washed with 350 *μ*l buffer BR3 as above, followed by two washes and spins with 500 *μ*l of buffer BR4, the later performed at maximum speed (16 000 **g**) for 3 min to dry the PAXgene column membrane. The columns were finally eluted with 40 *μ*l of buffer BR5 and the eluent collected in a 0.5 ml tube after a spin at 8000 **g** for 1 min. The final eluent was incubated for 5 min at 65°C in a heating block, then chilled on ice and the harvested RNA was stored at −80°C. qRT–PCR was then performed essentially as described in detail (Fong *et al*, 2000; McKinnon *et al*, 2002; Hu *et al*, 2003; Cummings *et al*, 2005). All the RT–PCR steps were carried out in an ABI Prism 7700 Sequence Detector, and quantitation was by the cycle threshold (CT) method.

## RESULTS AND DISCUSSION

### Method validation of the M65 Elisa assay

We have previously shown that the M30 assay yields a sigmoidal calibration curve of the type often associated with sandwich Elisa assays (Miller *et al*, 2001; Cummings *et al*, 2005). Here, we show that the M65 assay produces a linear calibration curve over the concentration range of 125–2000 U/l^−1^, with a mean regression correlation coefficient (*r*^2^) value of 0.996±0.003 (*n*=27). The linear response is believed to be due to the high affinity constants (10^−13^ M) of the two mouse monoclonal antibodies utilised, M6 and M5 (Kramer *et al*, 2004), for their respective CK18 epitopes and the subsequent faster reaction kinetics (Peter Björklund, PEVIVA, personal communication). Thus, the M65 Elisa assay only requires a 2 h incubation time period as compared to a 4 h incubation for M30 Apoptosense.

The M65 Elisa also proved to be highly reproducible with precision and accuracy (P and A) based on values obtained using the in kit QCs (200 and 1000 U l^−1^) (Table 1). Furthermore, precision and kit to kit reproducibility data were generated by use of independent external standard QCs consisting of both rCK18 and CK18 Asp^396^ NE added to human plasma (see Table 1). These studies were conducted over an 18-month period and were generated after running a total of 27 assays using 19 different kits, comprising three different production batches. Each experiment consisted of a seven point calibration curve run in duplicate, including a blank, while replicates numbers of each QC run per experiment ranged from *n*=3–8. Mean P and A achieved with the in kit QCs normally varied by <5% with the exception of accuracy for the low QC which varied by 8.3% (yielding a mean of 91.7% of that of its nominal value). This bias was introduced exclusively by kits from the earliest production batch, and appeared to be eliminated in the subsequent two production batches. Utilising the external standards, mean kit to kit variations in the concentration of antigen determined was 5.3% with rCK18 and 4.8% with CK18 Asp^396^ NE, normally well within the manufacturer's acceptance criteria of 10%, (http://web.peviva.se/index.asp).

Unlike the M30 Apoptosense Elisa that relies on caspase cleavage to reveal a neo-epitope mapped to positions 387–396 of CK18 (Ku *et al*, 1997; Leers *et al*, 1999), the M65 Elisa utilises the M6 antibody to capture and an HRP-conjugated M5 antibody to detect epitopes present in a domain common to the intact protein as well as the 21 kDa caspase cleaved fragment (see Figure 1) (Kramer *et al*, 2004). Although, intact CK18 is viewed as a stable insoluble structural component of the intermediate filaments (Fuchs and Weber, 1994), proliferating cells also possess a substantial pool of soluble CK18, which increases upon stress due to remodelling of cellular structure (Chou *et al*, 1993; Ku *et al*, 1997). Recently, cancer cells undergoing necrotic death have been shown to release intact CK18 in a manner analogous to the release of its caspase cleaved fragment from cells undergoing apoptosis (Kramer *et al*, 2004; Schutte *et al*, 2004). Thus, the M65 Elisa potentially detects both necrotic and apoptotic components, although it is unclear whether or not this assay is capable of detecting all presently known cell death mechanisms such as autophagy and mitotic catastrophe (Broker *et al*, 2005). Nevertheless, since both the M30 and M65 assays are calibrated with the same recombinant CK18 peptide fragment (CK18 Asp^396^ NE), their readouts are directly comparable and the ratio of the signal measured by each enables differentiation between cell death modes (Kramer *et al*, 2004). We have confirmed by use of our standard of caspase cleaved CK18 that both assays do indeed produce very similar results, normally within 10% of each other, whereas the M30 Apoptosense assay exhibits, as expected, very little cross reactivity for intact rCK18.

### Stability studies on the M30 and M65 antigens

**Figure 2:**
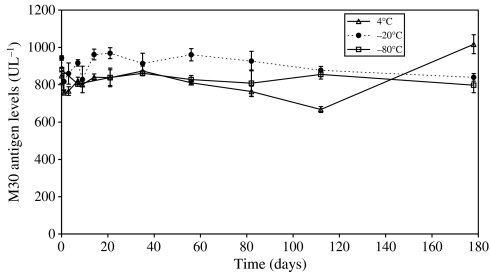
Stability of caspase cleaved CK18 (Asp^396^ neo-epitope, see Figure 1) in human volunteer plasma determined by the M30 Apoptosense assay. Each time point represents the mean value±standard deviation (s.d.) of *n*=3–8 replicates.

**Figure 3:**
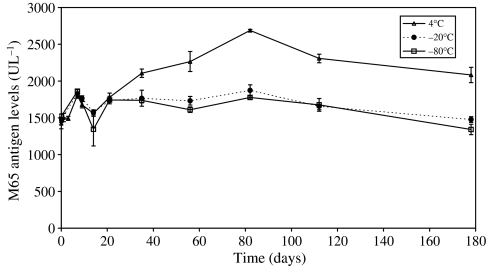
Stability of recombinant human cytokeratin 18 in human volunteer plasma determined by the M65 Elisa assay. Each time point represents the mean value±s.d. of *n*=3–8 replicates.

Long-term stability studies were conducted in order to establish the maximal period of time that human plasma could be stored at different temperature without *in situ* analyte degradation occurring. Six-month stability of both the M30 and M65 antigens in pooled volunteer plasma was studied at 4°C, −20°C and −80°C (Figures 2 and 3, respectively). In addition, plasma containing both M30 and M65 antigens were subjected to three cycles of freeze thawing at −80°C (data not shown).

The M30 antigen (CK18 Asp^396^ NE, see Figure 1) was shown to be stable over 6 months at both −20°C and −80°C (Figure 2), where instability is defined as a significant reduction by Students *t*-test in concentration between two consecutive time points. After 6 months incubation at −80°C instability was demonstrated at the 8 month time with an 11.1% reduction in value (*P*=<0.01) (data not shown). At 4°C clear fluctuations in concentration were recorded, whereby after 4 months a small but significant reduction in value was observed followed by a larger increase after 6 months.

A small but significant (*P*<0.01) reduction in concentration of full-length rCK18 occurred after 6 months at both −20°C and −80°C (10.9 and 19.9%, respectively) (Figure 3) indicating that the stability of the M65 antigen is restricted to only 4 months at these temperatures. Whereas at 4°C stability was only evident for 4 weeks, after which time a consistent rise in concentration occurred for up to 3 months before levels started to fall. Samples subjected to three cycles of freeze thawing had no deleterious effect on the concentration of both M30 and M65 antigens.

**Figure 4:**
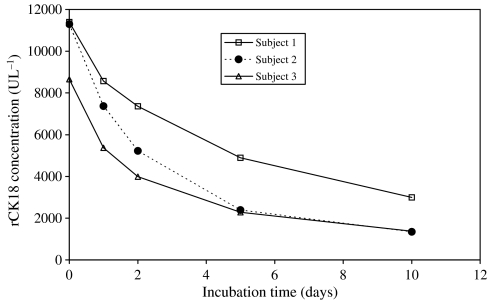
Stability of recombinant human CK18 in human volunteer plasma incubated at 37°C determined by the M65 Elisa assay. Each time point represents duplicate measurements. The mean half-life was 2.3 days.

Accelerated stability was also investigated at 37°C using individual volunteer plasma samples spiked at a higher concentration of rCK18 in order to accommodate for the more rapid decline in concentration (10 000 U l^−1^, Figure 4). In this case the aim was to address the issue of whether or not the M65 and M30 antigens are sufficiently stable in plasma at body temperatures to provide meaningful quantitative information on the kinetics of cell death occurring at distal sites in the body such as a tumour. Although the early stages of apoptosis occur within minutes, the final stages of lysosomal degradation can extend into hours, depending on cell type and tissue (Holdenrieder and Stieber, 2004a) and a lag period in the region 6–10 h has been reported before cleaved cytokeratin products are released from apoptotic bodies and necrotic cells (Einarsson and Barak, 1997; Schutte *et al*, 2004). Nonetheless, the stability studies conducted at 37°C yielded a mean half-life of 2.3 days for rCK18 in human plasma (Figure 4), compared to 3.8 days for CK18 Asp^396^ NE (Cummings *et al*, 2005).

The presence of different keratins in either plasma or serum of cancer patients has been recognised for many years and used as tumour markers in the diagnosis of cancer (Weber *et al*, 1984; Sundstrom and Stigbrand, 1994; Silen *et al*, 1995; Dobashi *et al*, 2000; Barak *et al*, 2004; Seregni *et al*, 2004). Therefore, it may be surprising that few publications have focused on the formal stability of these proteins as analytes in the systematic manner reported herein. Perhaps, in the case of keratins long term stability has always been presumed. Keratins are primarily responsible for providing cytoskeletal rigidity, and are now even believed to protect cells against chemical toxicity (Owens and Lane, 2003). Indeed, good physical and chemical stability has been demonstrated in a number of studies, especially in the case of the caspase cleaved fragments of CK18 (Ku *et al*, 1997; Carr, 2000; Aho, 2004). We have also confirmed that caspase cleaved CK18 is considerably more stable than the intact protein.

An unexpected finding of this work was that statistically significant increases in concentration of both intact CK18 and its caspase cleaved fragment occurred during the stability studies. A similar effect has also been observed in long--term stability of CK18 Asp^396^ NE in tissue culture media (Cummings *et al*, 2005). Investigations on keratin 8/18 breakdown and reorganisation during apoptosis have shown that the M30 antigen is released from apoptotic cells as large aggregates, which are believed to promote their stability in the circulation, including avoidance of uptake and clearance by the reticuloendothelial system (Schutte *et al*, 2004). It is likely that the CK18 Asp^396^ NE standard used in the present work, which was generated by the induction of apoptosis in cancer cells *in vitro*, also consists of aggregates. The possibility exits that with time these aggregates dissociate to reveal more epitope sites for antibody interaction thus giving the appearance of an increase in concentration. Cytokeratin 18 also has a strong propensity to form higher complexes both with keratin and non-keratin proteins (Liao and Omary, 1996; Hofmann and Franke, 1997; Coulombe *et al*, 1998) and can be detected in the circulation as a range of small and large polymeric protein complexes referred to as tissue polypeptide-specific antigen (TPS) (Rydlander *et al*, 1996). Therefore, as with the M30 antigen, it is conceivable that association occurs *in situ* when rCK18 is added to plasma and that with time these complexes also slowly disassociate, before the onset of proteolytic destruction or chemical degradation. Alternatively, in the case of intact rCK18 protein unfolding could occur with time and that might increase the accessibility of the detection antibody (M5) for its epitope. Possible explanations for the increase in concentration are currently under investigation.

### Within-day and between-day variability in baseline values of M30 and M65 antigens in cancer patients

**Figure 5:**
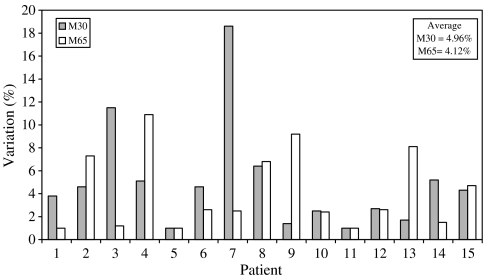
Within-day variations in baseline, predose values of M30 and M65 antigens in plasma of ovarian cancer patients before treatment with Carboplatin. Two separate blood samples were obtained with a 1–8 h gap between collections. The percentage difference in the two results for each patient is presented in the graph. In these studies the M65 assay was used to determine the M65 antigen, while the M30 Apoptosense assay was used to determine the M30 antigen.

**Figure 6:**
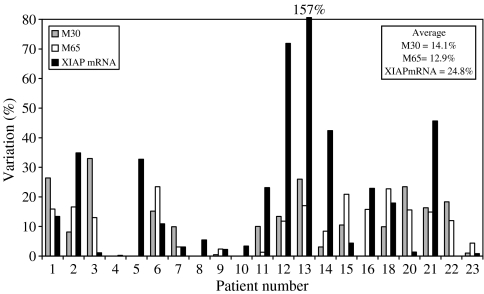
Between-day variations in baseline, predose plasma values of M30 and M65 antigens and XIAP mRNA measured in PBMCs by qRT–PCR. Two separate blood samples were obtained from patients entered into the AEG35156 phase I with a 5- to 7-day gap between collections. The percentage difference in the two results for each patient is presented in the graph. In these studies the M65 assay was used to determine the M65 antigen, while the M30 Apoptosense assay was used to determine the M30 antigen.

Within-day variations in the baseline levels of circulating M30 and M65 antigens detected in a cohort of 15 ovarian cancer patients are presented in Figure 5, while between-day variations obtained by analysis of two predose samples collected 5–7 days apart from patients entered into the AEG35156 trial are shown in Figure 6. In these studies, the M65 assay was used to determine M65 antigen, while the M30 Apoptosense assay was used to determine the M30 antigen. For within-day, the percentage differences measured (5.0% average for M30 and 4.1% average for M65) were within, or at least close to, the normal levels of variability associated with the methods indicating a comparatively small contribution from the biomarker itself. These data are in contrast to many typical plasma PD assays which are often associated with 30% or greater imprecision (Miller *et al*, 2001; Lee *et al*, 2005).

In the case of between-day (Figure 6), a much greater level of variation was detected, as might be expected, which clearly included a component due to biological variability. Nonetheless, the mean variation was 14.1% for M30 and 12.9% for M65 and only in one example (M30 for patient 3) did this value exceed 30% (Miller *et al*, 2001; Lee *et al*, 2005).

Traditionally, detection of a keratin antigen in serum or plasma, such as tissue polypeptide antigen (TPA, fragments of CK8, 18 and 19), TPS (CK18 fragments and complexes) and CYFRA 21-1 (caspase cleaved CK19), has been viewed as a marker of bulk tumour burden (Barak *et al*, 2004; Seregni *et al*, 2004) while, some reports have suggested that they might also predict treatment outcome and overall survival (Einarsson and Barak, 1997; Takada *et al*, 2004). However, it is now believed that the presence of M30, M65 and TPS antigens in the circulation may be more indicative of active processes taking place in the tumour such as apoptosis or other forms of cell death (Einarsson and Barak, 1997; Ueno *et al*, 2003; Kramer *et al*, 2004; Schutte *et al*, 2004). Indeed, other keratin markers such as TPA and CYFRA21-1, which consist of caspase cleaved fragments, are now also being proposed to be markers of apoptosis rather than tumour volume (Dohmoto *et al*, 2001; Kramer *et al*, 2004). Thus, the between-day fluctuations observed in the present study in baseline levels of M30 and M65 are more likely to be illustrative of tumour growth dynamics reflecting the balance between cellular proliferation and attrition due to cell death (Evan and Vousden, 2001; Lowe *et al*, 2004).

The ideal circulating surrogate cancer PD biomarker assay would be specific for tumour cells (Levenson, 2004; Lee *et al*, 2005). M30 and M65 Elisa's do show a higher degree of specificity compared to other surrogate markers of cell death such as serum nucleosomal DNA Elisa (nDNA) (Holdenrieder and Stieber, 2004a, 2004b), in that they are not subject to interference by bone marrow cell death. Notwithstanding, the M30 and M65 Elisa's would not be capable of distinguishing between circulating CK18 forms arising from normal tissue cell death as compared to tumour cell death. A spate of recent publications have reported that the M30 assay will detect an increased signal in the serum of patients with chronic hepatitis, liver toxicity due to viral infection and critical illness due to sepsis (Bantel *et al*, 2004; Roth *et al*, 2004; Kronenberger *et al*, 2005). These medical conditions, at least in an attenuated form, may also be present in cancer patients due to issues such as general state of health, presence of viral infections and morbidity caused by the toxic side effects of therapy. Nonetheless, in the present study over a 5–7 days period in a group of 23 patients variations in the baseline levels of the M30 and M65 assays did not normally exceed 30%, whereas by contrast in sepsis a 400% increase in circulating M30 was detected (Roth *et al*, 2004), in fibrotic liver injury and chronic hepatitis a 250% increase occurred (Kronenberger *et al*, 2005) and after treatment with estamustine/vinorelbine chemotherapy a greater than 200% increase has been reported (Kramer *et al*, 2004). Indeed, taking a two-fold increase in signal over mean noise as significant (Kramer *et al*, 2004), this would translate into an increase of >28% for M30 and >26% for M65 being characterised as a positive result. Clearly the M30 and M65 have considerable spare dynamic range to record even a modest effect on tumour cell death/apoptosis.

### Predose studies in cancer patients on the baseline values and variability in XIAP mRNA expression determined by qRT–PCR

The graph of between-day variations in predose baseline levels of XIAP mRNA in peripheral blood mononuclear cells (PBMCs) is to found in Figure 6. Here, the mean variation was established to be 25%, ranging from 0.2 to 157%. These values are higher than for the M30 and M65 and this was surprising, for two reasons. First, although qRT–PCR is only considered a quasiquantitative method, since it lacks calibration with an authentic standard, it nevertheless generates numerical results, with a high degree of precision, normally less than 5% and often exceeding the M30 and M65 Elisa's in performance (Dumur *et al*, 2002; Jones *et al*, 2003; Cummings *et al*, 2005). Second, qRT–PCR of XIAP mRNA should be subject to fewer potential variables than the M30 and M65 assays (see above). Thus, we believe that the high degree of variability observed in Figure 6 is probably due to genuine between-day fluctuations in XIAP mRNA expression in the two predose PBMC samples collected from the different cancer patients. To the best of our knowledge, no similar study has been published addressing the issue of biomarker variability in XIAP mRNA levels in PBMCs in cancer patients or normal volunteers. The differences we observed may possibly be explained either by changes in leucocyte populations expressing varying amounts of XIAP, and/or increases in cellular XIAP levels due to cell division or stimulation with G-CSF or GM-CSF. Both these cytokines have been reported to induce XIAP in leucocytes or AML cells at the protein level (Lin *et al*, 2002; Zhang *et al*, 2002; O’Neill *et al*, 2004).

The measurement of XIAP mRNA expression in PBMCs represents the primary PD assay being utilised in the AEG35156 trial (Cummings *et al*, 2005). Current thinking suggests that a total knockout of XIAP in tumour would not necessarily be required rather a partial knockdown may be sufficient to lower the apoptotic threshold to take advantage of the intrinsic proapoptotic signalling cascades presumed to be operative in many cancers (Kaufmann and Vaux, 2003; Reed, 2003; Wright and Duckett, 2005). Therefore, only a 50% reduction may be sufficient to produce a therapeutic effect (Lacasse *et al*, 2005). Returning to the 2 × signal to noise rule, in the case of the present qRT–PCR method, a knockdown of greater than 50% would be required to be considered a valid result, although even here one must be aware that extreme outliers are not uncommon (Figure 6). To counteract this problem a potential solution may be to conduct repeated and relatively frequent determinations of XIAP mRNA levels during therapy where a consistent trend may emerge that can then be more confidently distinguished from background variability.

It should be noted that the number of patients employed in both trials to evaluate variability in baseline values of M30 and M65 antigens and XIAP mRNA are based on typical phase I trial numbers (15–23). The present studies are intended as a first stage, and have yielded encouraging results. We are in the process of conducting further and larger clinical studies utilising the M30 and M65 assays as biomarkers of tumour cell death, not just from the point of view of method validation and baseline studies, but eventually to provide definitive proof or otherwise as to the clinical validity and utility of M30 and M65 as surrogate PD biomarkers of tumour cell death – biomarker qualification (Lee *et al*, 2005).

In summary, data are presented demonstrating that the M65 Elisa is a highly reproducible assay for the determination of CK18 and CK18 Asp^396^ NE in human plasma. The stability studies have revealed that caspase cleaved CK18 is more stable than the intact protein and that long-term storage of samples is not recommended for more than 4 months even at −20°C and −80°C. Finally, we show in patient studies that the typical baseline variation in the analytes of the M30 and M65 Elisa's and a qRT–PCR method for XIAP mRNA is comparatively small at 14, 13 and 25%, respectively.

## Figures and Tables

**Table 1:** Reproducibility of the M65 Elisa assay

**QC standard**	**Mean accuracy (%)**	**Mean precision (%)**	**Number of assays**
Low In kit QC (200 U l^−1^)	91.7 (range 73.1–109.7)	1.0 (range 0.8–16.7)	27
High In Kit QC (1000 U l^−1^)	102.5 (range 95.9–115.4)	2.9 (range 0.3–9.8)	27
			
	**Mean precision (%)**	**Kit to kit CV (%)**	**Number of kits**
rCK18 (circa 1500 U l^−1^)	2.9 (range 1.3–6.6)	5.3 (range 1.5–11.6)	8
CK18 Asp^396^ NE (circa 800 U l^−1^)	2.0 (range 0.6–3.1)	4.8 (range 2.3–7.3)	4

Abbreviations: CK18=cytokeratin 18; QC=quality controls.
